# Effects of integrase inhibitor-based antiretroviral therapy on brain outcomes according to time since acquisition of HIV-1 infection

**DOI:** 10.1038/s41598-021-90678-6

**Published:** 2021-05-28

**Authors:** Anna Prats, Ignacio Martínez-Zalacaín, Beatriz Mothe, Eugènia Negredo, Núria Pérez-Álvarez, Maite Garolera, Sira Domènech-Puigcerver, Pep Coll, Michael Meulbroek, Anna Chamorro, Carmina R. Fumaz, Maria J. Ferrer, Bonaventura Clotet, Carles Soriano-Mas, Jose A. Muñoz-Moreno

**Affiliations:** 1 Fundació Lluita Contra La SIDA (FLS) - Hospital Universitari Germans Trias I Pujol, Ctra. del Canyet, 08916 Badalona, Barcelona, Catalonia Spain; 2 Unitat de Parkinson I Trastorns del Moviment, Clínica Teknon Grupo Quirón-Salud, Barcelona, Catalonia Spain; 3 Departament de Psiquiatria I Medicina Legal, Universitat Autònoma de Barcelona (UAB), Cerdanyola del Vallès, Catalonia Spain; 4 Institut D’Investigació Biomèdica de Bellvitge (IDIBELL) - Hospital Universitari de Bellvitge, Feixa Llarga, L’Hospitalet de Llobregat, 08907 Barcelona, Catalonia Spain; 5 Departament de Ciències Clíniques, Universitat de Barcelona (UB), Barcelona, Catalonia Spain; 6 IrsiCaixa - Institut de Recerca de La SIDA, Hospital Universitari Germans Trias I Pujol, Badalona, Catalonia Spain; 7 Universitat de Vic - Universitat Central de Catalunya (UVic-UCC), Vic, Catalonia Spain; 8 Departament D’Estadística I Investigació Operativa, Universitat Politècnica de Catalunya (UPC), Barcelona, Catalonia Spain; 9 Consorci Sanitari Hospital de Terrassa, Terrassa, Catalonia Spain; 10 Grup de Recerca Consolidat en Neuropsicologia, Universitat de Barcelona (UB), Barcelona, Catalonia Spain; 11 Institut de Diagnòstic Per La Imatge (IDI) - Hospital Universitari Germans Trias I Pujol, Badalona, Catalonia Spain; 12 Projecte dels NOMS - Hispanosida, BCN Checkpoint, Barcelona, Catalonia Spain; 13 CIBER Salud Mental (CIBERSAM), Instituto de Salud Carlos III, Barcelona, Catalonia Spain; 14 Departament de Psicobiologia i Metodologia de Les Ciències de La Salut, Universitat Autònoma de Barcelona (UAB), Cerdanyola del Vallès, Catalonia Spain; 15 Facultat de Psicologia I Ciències de L’Educació, Universitat Oberta de Catalunya (UOC), Barcelona, Catalonia Spain

**Keywords:** Infectious diseases, Cognitive neuroscience, Anxiety, Depression, Central nervous system infections

## Abstract

Integrase strand transfer inhibitors (INSTI) are a main component of the current antiretroviral regimens recommended for treatment of HIV infection. However, little is known about the impact of INSTI on neurocognition and neuroimaging. We developed a prospective observational trial to evaluate the effects of INSTI-based antiretroviral therapy on comprehensive brain outcomes (cognitive, functional, and imaging) according to the time since HIV-1 acquisition. We recruited men living with HIV who initiated antiretroviral therapy with INSTI < 3 months since the estimated date of HIV-1 acquisition (n = 12) and > 6 months since estimated date of HIV-1 acquisition (n = 15). We also recruited a group of matched seronegative individuals (n = 15). Assessments were performed at baseline (before initiation of therapy in HIV arms) and at weeks 4 and 48. Baseline cognitive functioning was comparable between the arms. At week 48, we did not find cognitive differences between starting therapy with INSTI earlier than 3 months or later than 6 months after acquisition of HIV-1 infection. Functional status was poorer in individuals diagnosed earlier. This effect recovered 48 weeks after initiation of therapy. Regarding brain imaging, we found that men living with HIV initiating antiretroviral therapy later experienced a greater decrease in medial orbitofrontal cortex over time, with expected negative repercussions for decision-making tasks.

## Introduction

Cognitive disorders are a common complication in people living with HIV (PLWH). HIV-associated neurocognitive disorders (HAND) have been described in 30–50% of this population, despite the use of combination antiretroviral therapy (cART)^1,2^. The neuropathogenesis of HAND remains unclear, although it appears to be multifactorial. Early HIV-related events before initiation of antiretroviral therapy could induce irreversible damage to the central nervous system (CNS). Prompt initiation of cART could therefore prevent or mitigate the effects of HIV on the CNS^3–5^. This possibility is supported by recent data, which show attenuation of brain inflammatory processes after initiation of cART during acute or primary infection^6–9^.

Integrase strand transfer inhibitors (INSTI) are currently part of the first-line cART regimens recommended for HIV treatment^10^. However, their impact on the CNS remains controversial. Most of the studies investigating the CNS effects of INSTI have evaluated the potential appearance of neuropsychiatric symptoms, notably missing other crucial CNS dimensions, such as cognitive performance, functional outcomes, and brain imaging^11^. One recent cross-sectional study provided information on the outcomes of cognitive and structural brain imaging^12^. Importantly, the authors found poorer performance in the verbal learning and memory domain in PLWH on INSTI-based regimens compared with PLWH not taking INSTI and revealed smaller volumes in the frontal, brainstem, and cerebellar regions.

We developed a prospective observational trial to evaluate the impact of INSTI on comprehensive brain outcomes and investigated the association with time since acquisition of HIV-1. We recruited men living with HIV (MLWH) who started antiretroviral therapy with INSTI < 3 months after the estimated date of acquisition, MLWH who initiated therapy with INSTI > 6 months after the estimated date of acquisition, and a group of matched seronegative people.

## Methods

### Study participants and design

Thirty cART-naïve HIV-1-positive adult men were recruited at the HIV Clinical Unit of Germans Trias i Pujol University Hospital, Barcelona, Catalonia, Spain, from October 2015 to April 2017. The participants were ≥ 18 years old and were expected to initiate cART including INSTI. Therapy was initiated following clinical antiretroviral guidelines.

MLWH were divided into 2 groups according to the time since estimated date of HIV-1 acquisition. The first arm (early treatment arm) met the following criteria: recent or acute infection confirmed by (1) positive plasma viral load and/or presence of p24 antigen with a negative enzyme-linked immunosorbent assay (ELISA) result, or (2) positive ELISA result and undefined Western blot result, or (3) positive ELISA result and absence of antigen band p31 in a positive Western blot test, or (4) seroconversion according to ELISA in less than 3 months; and agreement to start cART in less than 90 days from the estimated date of acquisition of HIV-1. The second arm (later treatment arm) comprised participants with HIV who did not meet the criteria for the early treatment arm and whose estimated time since acquisition was longer than 6 months, as confirmed by viral load set-point criteria after 2 consecutive viral loads with a determination on the same logarithmic scale and agreement to start cART 6 months after the estimated date of acquisition. A control group matched for age, sex, and educational level with the HIV arms and comprising non-MLWH was recruited from a community health center. The exclusion criteria were age > 60 years, intellectual disability, current or past neurological disease, current or past CNS-related opportunistic infection, and current severe psychiatric disorder. MLWH who presented poor adherence (< 90%), interrupted cART, or ceased INSTI during follow-up were excluded from the study analyses. Study assessments were performed at baseline (i.e., before initiation of cART in HIV arms) and at weeks 4 and 48 after initiation of cART.

All participants gave their informed consent before enrolment. The study was approved by the Research Ethics Committee of Germans Trias i Pujol University Hospital (*FLS-ANT-2015–01*) and was conducted in compliance with the Helsinki Declaration of 1964 (1996 revision) and Good Clinical Practice guidelines. The trial is registered at ClinicalTrials.gov (*NCT03835546, date of registration: 08/02/2019*).

### Cognitive assessment

Participants underwent a comprehensive neuropsychological assessment of 6 cognitive domains, with 2 measures per domain (Supplementary Table S1). A total of the 12 scores obtained made it possible to calculate a global cognitive measure, the neuropsychological *z* score (NPZ-12), which was based on the average of standardized *z* scores. Cognitive impairment was defined as performing ≥ 1 standard deviation below the normative mean in ≥ 2 of the cognitive domains assessed. Standardized *z* scores were always used for comparisons. Cognitive complaints were also recorded based on the European AIDS Clinical Society guidelines^13^. HAND were determined based on the Frascati criteria^14^.

### Functional assessment

Functional outcomes included CNS adverse events, daily functioning, emotional status, and quality of life. CNS adverse events were assessed by an adapted inventory including adverse effects from the US Food and Drug Administration (FDA) for the different INSTI studied. Daily functioning was evaluated using a version of the Instrumental Activities of Daily Living (IADL) questionnaire^15,16^. Depressive and anxiety symptoms were recorded using the Hospital Anxiety and Depression Scale (HADS)^17,18^. The Perceived Stress Scale (PSS-10) was used for perceived stress^19,20^. Quality of life was evaluated using an adapted version of the Medical Outcomes Study HIV Health Survey (MOS-HIV) questionnaire^21^.

### Brain imaging acquisition and preprocessing

Brain imaging data were collected using a 3 T magnetic resonance imaging scanner (Siemens Verio, Siemens Healthcare Sector, Erlangen, Germany) equipped with a 32-phased-array head coil offered by the manufacturer. A high-resolution T1-weighted 3D structural image was acquired individually for each participant in the axial plane at the 3 study timepoints with the following parameters: 192 slices; repetition time = 1900 ms; echo time = 2.72 ms; flip angle = 9$^\circ$; field of view = 260 × 260 mm; matrix size = 256 × 256 pixels; in-plane resolution = 0.96 × 0.96 mm^2^; slice thickness = 0.9 mm. Data were preprocessed executing technical computing software (MATLAB 7.14; The MathWorks, Natick, MA, USA) and statistical parametric mapping (SPM12; The Welcome Department of Imaging Neuroscience, London, UK). Images were examined by experienced members of the research team, mainly to rule out presence of artifacts or other effects that could interfere in data analyses. Subsequently, images were preprocessed using 2 independent approaches performed in parallel. First, at the individual level, longitudinal preprocessing consisted of an initial rigid-body within-subject co-registration to the first scan to ensure good starting estimates. This was followed by a pairwise longitudinal registration of the scans for each participant to obtain an average image and a Jacobian difference map^22^. The average image was then segmented, and the gray matter voxels were multiplied by the Jacobian difference map to obtain a gray matter volume change map for each participant^23^. Next, we generated a specific template of our study sample in Montreal Neurological Institute (MNI) space using a Diffeomorphic Anatomical Registration Through Exponentiated Lie Algebra algorithm (DARTEL)^24^, which was used to spatially normalize the gray matter volume change maps.

### Data analyses

Data analyses were based on cross-sectional and prospective comparisons from baseline to week 4 and from baseline to week 48. Descriptive results were based on comparisons between the 3 study arms. Prospective results were based on 2-arm comparisons (early treatment arm vs the control arm; and later treatment arm vs the control arm).

The primary study endpoint was change in neurocognitive performance from baseline to week 48 measured based on the NPZ-12 score. Secondary endpoints comprised the change in neurocognitive performance from baseline to week 4 measured based on the NPZ-12 score, as well as the change in specific cognitive scores, functional outcomes, and structural brain imaging markers from baseline to week 4 and from baseline to week 48. Changes were compared between each HIV arm and the control group (2-arm comparisons).

Statistical tests were applied according to the type and distribution of variables and included the chi-square, Fisher, independent *t*, and ANOVA for mean tests. Longitudinal repeated-measures ANOVA was used for prospective intra-group comparisons. Linear mixed models were applied to study the effect of unbalanced variables at baseline. All comparisons were univariate and 2-tailed. Statistical significance was set at *p* < 0.05. Effect sizes were also calculated to quantify the magnitude of the differences found using Cohen's *d*. Values were considered small when scores were less than 0.20, medium when they ranged between 0.50 and 0.80, and large when they were over 0.80. Power calculations were performed for the primary study endpoint. With a power of 80%, the effect size cut-off was established at 0.55. Statistical analyses were performed using SPSS, version 15 (SPSS Inc, Chicago, Illinois, USA) and R, version 3.5.1 (https://cran.r-project.org).

Brain imaging data were analyzed with SPM12. For the longitudinal analyses, gray matter volume change maps were compared to detect areas of increase or decrease in volume across the different timepoints. Baseline gray matter images were also compared to detect putative voxel-wise volume differences at baseline across the 3 arms. Gray matter images from each timepoint were used to extract the volumes of the regions of interest stemming from previous analyses, which were further studied in SPSS. SPM12 analyses were thresholded at *p* < 0.05, and the family wise error (FWE) corrected for the number of independent comparisons.

## Results

### Characteristics of the study participants

A total of 45 participants were recruited, 15 per study arm. Three (6%) of those in the early treatment arm were excluded due to protocol failure: 2 because the estimated date since HIV-1 acquisition could not be established, and 1 because an intellectual disability was detected. One participant (2%) in the control arm was not included in the data analyses at week 4 because of loss to follow-up. Eight participants (19%) were excluded from the week-48 analyses: in the early treatment arm, 1 (2%) was lost to follow-up; in the later treatment arm, 2 (5%) interrupted cART voluntarily and 1 (2%) switched antiretroviral therapy due to a lipid-related issue; and in the control arm, 2 (5%) were lost to follow-up and 2 (5%) initiated pre-exposure prophylaxis (PrEP).

Regarding the characteristics of the sample, participants had a mean (standard deviation [SD]) age of 33 (10) years and a mean of 14 (3) years of education. We detected significant differences in days from diagnosis of HIV-1 infection to initiation of cART (*p* = 0.028) and plasma viral load log_10_ (*p* = 0.010). The remaining demographic and clinical variables were equivalent between the groups. INSTI were distributed as follows: dolutegravir, 12 participants (44%); elvitegravir, 10 (37%); and raltegravir, 5 (19%) (*p* = 0.198). Table 1 shows the characteristics of the study sample.

**Table 1:** Characteristics of the study sample.

	Early treatment (n = 12)	Later treatment (n = 15)	Control (n = 15)	*p* value
Age, years	34 (10)	31 (9)	32 (10)	0.724
Male, n (%)	12 (100)	15 (100)	15 (100)	–
Years of education	14 (2)	13 (2)	13 (3)	0.881
Estimated days since HIV-1 acquisition to cART initiation	63 (20)	–	–	–
Days since HIV diagnosis to cART initiation	18 (12)	72 (80)	–	**0.028**
cART naïve (%)	12 (100)	15 (100)	–	–
INSTI-based therapy initiated, n (%)				0.198
TDF + FTC + RAL	4 (33)	1 (7)	–	–
TDF/TAF + FTC + EVG	4 (33)	6 (40)	–	–
ABC + 3TC + DTG	4 (33)	8 (53)	–	–
Plasma viral load log_10_^a^	5.1 (0.97)	4.1 (0.88)	–	**0.010**
CD4 cell count	537 (239)	475 (203)	–	0.479
CD8 cell count	1025 (667)	1072 (839)	–	0.877
CD4/CD8 ratio	0.64 (0.38)	0.53 (0.18)	–	0.350
Contributing comorbidities^b^, n (%)	6 (50)	5 (33)	5 (33)	0.604
Illicit drug use (last month), n (%)	6 (50)	5 (33)	4 (27)	0.441
Marijuana	5 (42)	4 (27)	2 (13)	0.250
Club drugs^c^	2 (17)	3 (20)	2 (13)	0. 887

Values are expressed as mean (standard deviation) except when indicated otherwise.

Continuous variables were compared using the ANOVA for means test. Categorical variables were compared using the chi-square test. Statistical significance was set at *p* < 0.05. Bold values denote statistical significance.

Abbreviations: 3TC = lamivudine; ABC = abacavir; cART = combination antiretroviral therapy; DTG = dolutegravir; EVG = elvitegravir; FTC = emtricitabine; INSTI = integrase strand transfer inhibitor; TAF = tenofovir alafenamide fumarate; TDF = tenofovir disoproxil fumarate; RAL = raltegravir.

^a^Detection limit of ≤ 40 copies/mL.

^b^Based on the Frascati criteria (Antinori A, et al., Neurology, 2007), which refer to pre-existing conditions that may act as contributors in cognitive performance, mainly mild psychiatric disorder, psychopharmacological treatment, illicit drug use, and alcohol consumption.

^c^Defined as stated by the National Institute on Drug Abuse (NIDA) from the National Institutes of Health (NIH). Club drugs are those that tend to be used in bars, nightclubs, concerts, and parties and include GHB, Rohypnol®, ketamine, MDMA (ecstasy), methamphetamine, and LSD (acid).

### Cognitive outcomes

**Figure 1:**
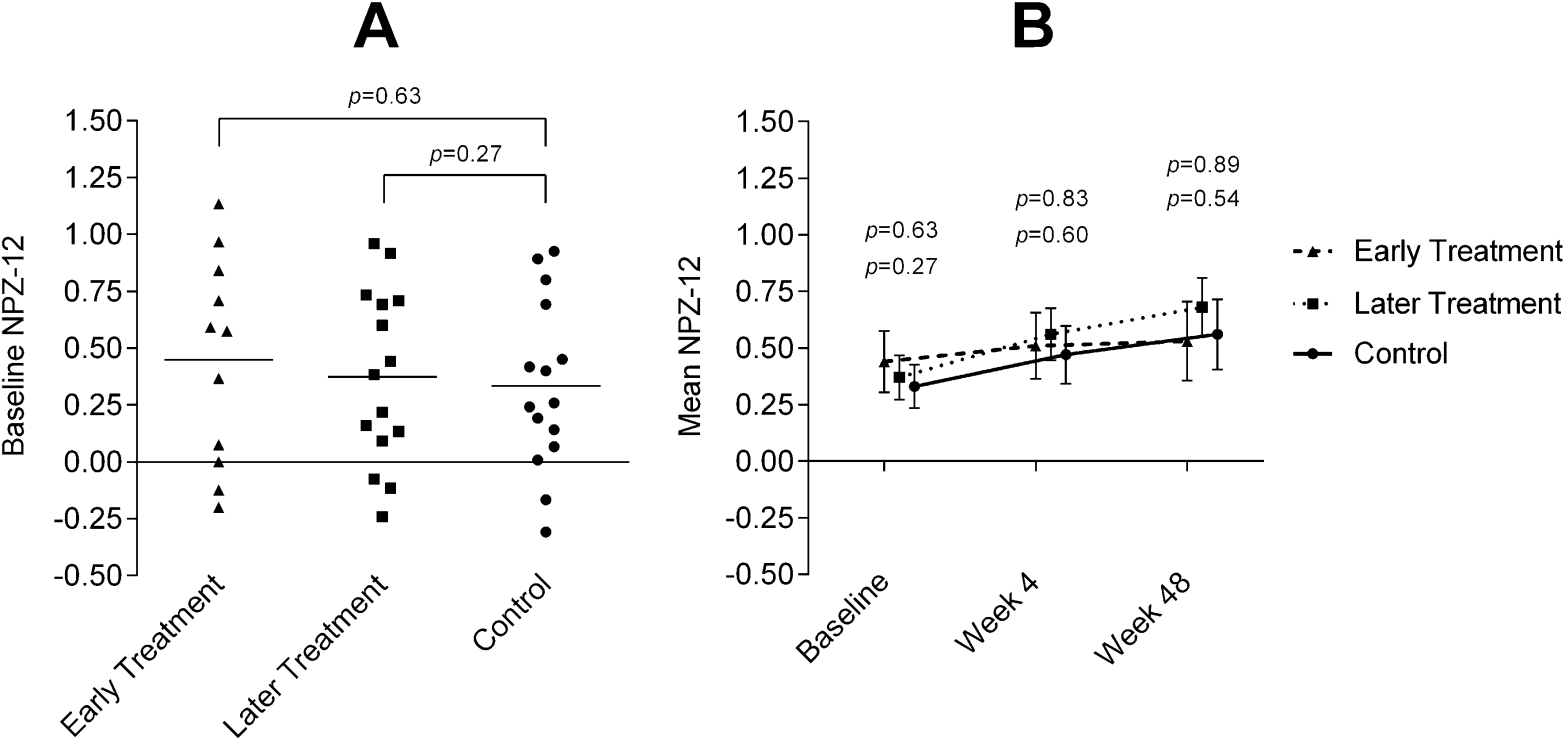
Baseline and progression of NPZ-12. (**A**) Baseline NPZ-12 scores. Horizontal bars represent the mean. The statistical test applied was ANOVA for means. (**B**) Mean NPZ-12 scores. The *p* values shown above indicate early treatment vs control comparisons. The *p* values shown below indicate later treatment vs control comparisons. The statistical test applied was ANOVA for means.

At baseline, no significant differences were found between the 3 arms in frequency of cognitive complaints (*p* = 0.962), frequency of cognitive impairment (*p* = 0.202), and global cognitive score (NPZ-12) (*p* = 0.771) (Fig. 1A). Similarly, no differences were found between specific cognitive scores (Supplementary Table S1). A worse NPZ-12 score was associated with a longer time since diagnosis of HIV-1 infection (*p* = 0.025) and a higher rate of potential comorbidities for cognitive impairment (*p* = 0.017).

Regarding the primary study endpoint, no differences were detected at week 48: early treatment arm, 0.08 (0.26); later treatment arm, 0.31 (0.28); control arm, 0.25 (0.19) (*p* = 0.147) (Fig. 1B). Likewise, no differences were found previously at week 4 (mean [SD] change in NPZ-12): early treatment arm, 0.06 (0.20); later treatment arm, 0.14 (0.25); control arm, 0.11 (0.28); *p* = 0.724. Equivalent results were confirmed when linear mixed models were applied, taking into consideration unbalanced variables at baseline (i.e., days since HIV diagnosis to cART initiation and plasma viral load log_10_). Changes in specific cognitive scores are shown in Supplementary Table S2.

### Functional outcomes

At baseline, depressive (*p* = 0.032), anxiety (*p* = 0.044), and perceived stress (*p* = 0.042) symptoms were significantly higher in the early treatment arm. The remaining functional outcomes were comparable (Supplementary Table S3).

**Figure 2:**
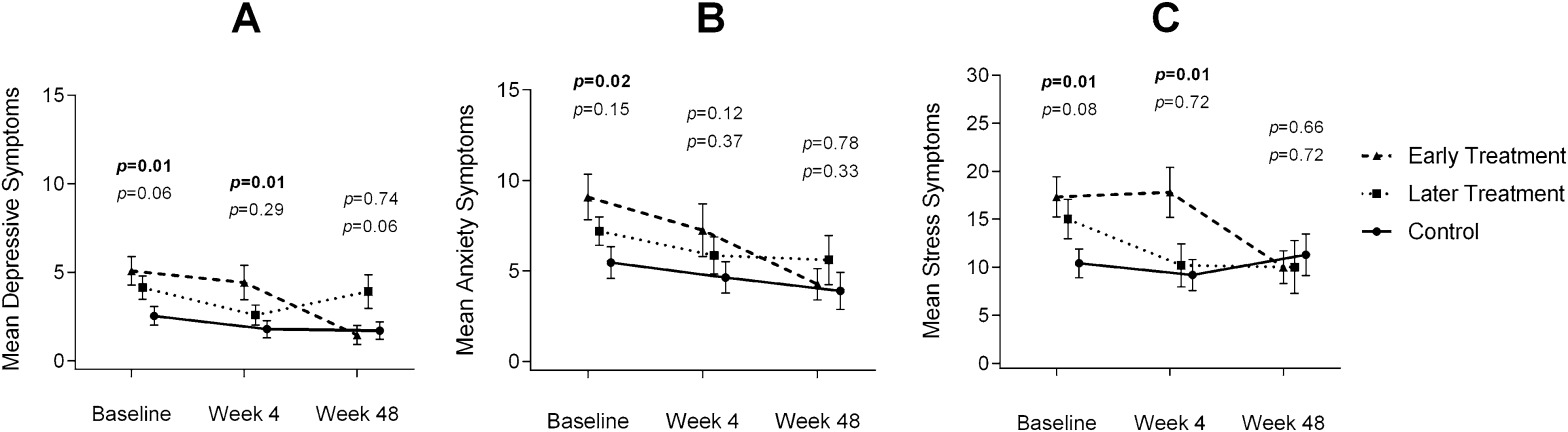
Progression of depressive, anxiety, and stress symptoms. The *p* values shown above indicate early treatment vs control comparisons. The *p* values shown below indicate later treatment vs control comparisons. The statistical test applied was ANOVA for means. Bold *p* values denote statistical significance. (**A**) Mean depression scores. Bars represent the standard error of the mean. (**B**) Mean anxiety scores. Bars represent the standard error of the mean. (**C**) Mean stress scores. Bars represent the standard error of the mean.

At week 4, a more marked change was observed in daily functioning in the early treatment arm than in the control arm: 1.25 (1.65) vs 0 (1.30); *p* = 0.047, *d* = 0.82 (Table 2). This change was not maintained at week 48. A significantly negative change was found in depression, anxiety, and perceived stress symptoms at week 48 in the early treatment arm compared with the control arm, with similar emotional status in both groups: depressive, –3.55 (2.58) vs –0.60 (1.43), *p* = 0.005, *d* = –1.34; anxiety, –5.18 (3.46) vs –1.30 (2.49), *p* = 0.009, *d* = –1.23; perceived stress, –7.55 (3.88) vs 1.40 (4.72), *p* < 0.001, *d* = –2. In the later treatment arm, symptoms of stress had improved at week 48: –6.50 (9.24) vs 1.40 (4.72), *p* = 0.024, *d* = –1.01. Figures 2A–C summarize the progression of depressive, anxiety, and stress symptoms respectively.

**Table 2:** Change in cognitive and functional outcomes.

Change from baseline	Early treatment (n = 12)		Later treatment (n = 15)		Control (n = 15)		*p* value^a^^;^^b^	*d* size^a^^;^^b^
	No. of subjects	Mean (SD)	No. of subjects	Mean (SD)	No. of subjects	Mean (SD)		
**Global**								
NPZ-12								
Week 4	12	0.06 (0.20)	15	0.14 (0.25)	14	0.11 (0.28)	0.602; 0.789	−0.24; 0.07
Week 48	11	0.08 (0.26)	15	0.31 (0.28)	10	0.25 (0.19)	0.117; 0.538	−0.17; 0.25
**CNS adverse events**								
Total								
Week 4	12	0.83 (6.38)	14	−0.35 (5.87)	14	−0.57 (5.82)	0.563; 0.924	0.22; −0.04
Week 48	11	−2.90 (8.76)	12	0.33 (7.58)	10	0.30 (5.77)	0.340; 0.991	−0,41; −0.00
**Daily functioning**								
Total impaired areas								
Week 4	12	1.25 (1.65)	14	−0.28 (1.26)	14	0 (1.30)	**0.047**; 0.561	0.82; −0.21
Week 48	11	0.54 (1.69)	12	0.33 (2.01)	10	0.30 (0.82)	0.683; 0.962	0.17; 0.02
**Emotional status**								
Depressive symptoms								
Week 4	12	−0.67 (2.80)	14	−1.50 (3.15)	14	−0.71 (1.68)	0.960; 0.419	0.02; −0.30
Week 48	11	−3.55 (2.58)	12	−0.50 (3.84)	10	−0.60 (1.43)	**0.005**; 0.935	−1.34; 0.03
Anxiety symptoms								
Week 4	12	−1.83 (3.97)	14	−1.43 (2.44)	14	−0.57 (1.55)	0.319; 0.278	−0.42; −0.41
Week 48	11	−5.18 (3.46)	12	−2.00 (2.93)	10	−1.30 (2.49)	**0.009**; 0.557	−1.23; −0.25
Perceived stress								
Week 4	12	0.50 (8.36)	14	−5.36 (9.14)	14	−0.71 (3.95)	0.632; 0.093	0.18; −0.64
Week 48	11	−7.55 (3.88)	12	−6.50 (9.24)	10	1.40 (4.72)	**0.001**; **0.024**	−2.0; −1.01
**Quality of life**								
Global dimension								
Week 4	12	−0.16 (0.71)	14	0.07 (0.47)	14	0.07 (0.61)	0.815; 1	−0.34; 0.00
Week 48	11	0.18 (0.40)	12	0.08 (0.51)	10	0.10 (0.73)	0.753; 0.953	0.15; −0.03

Values are expressed as mean (standard deviation).

For global neurocognitive performance (NPZ-12) and quality of life, higher change scores represent improvement.

For CNS adverse events, daily functioning, emotional status, and stress, lower change scores represent improvement.

The treatment and control arms were compared using the independent *t* test. Statistical significance was set at *p* < 0.05. Bold values denote statistical significance.

*d* size was obtained using Cohen's *d*. Values are considered small when < 0.20, medium when ≥ 0.50 and ≤ 0.80, and large when > 0.80.

Abbreviations: CNS = central nervous system.

^a^Comparison between early treatment and control arms.

^b^Comparison between later treatment and control arms.

### Brain imaging outcomes

No differences across the study groups were found in the baseline structural brain imaging assessments (*p* = 0.449).

**Figure 3:**
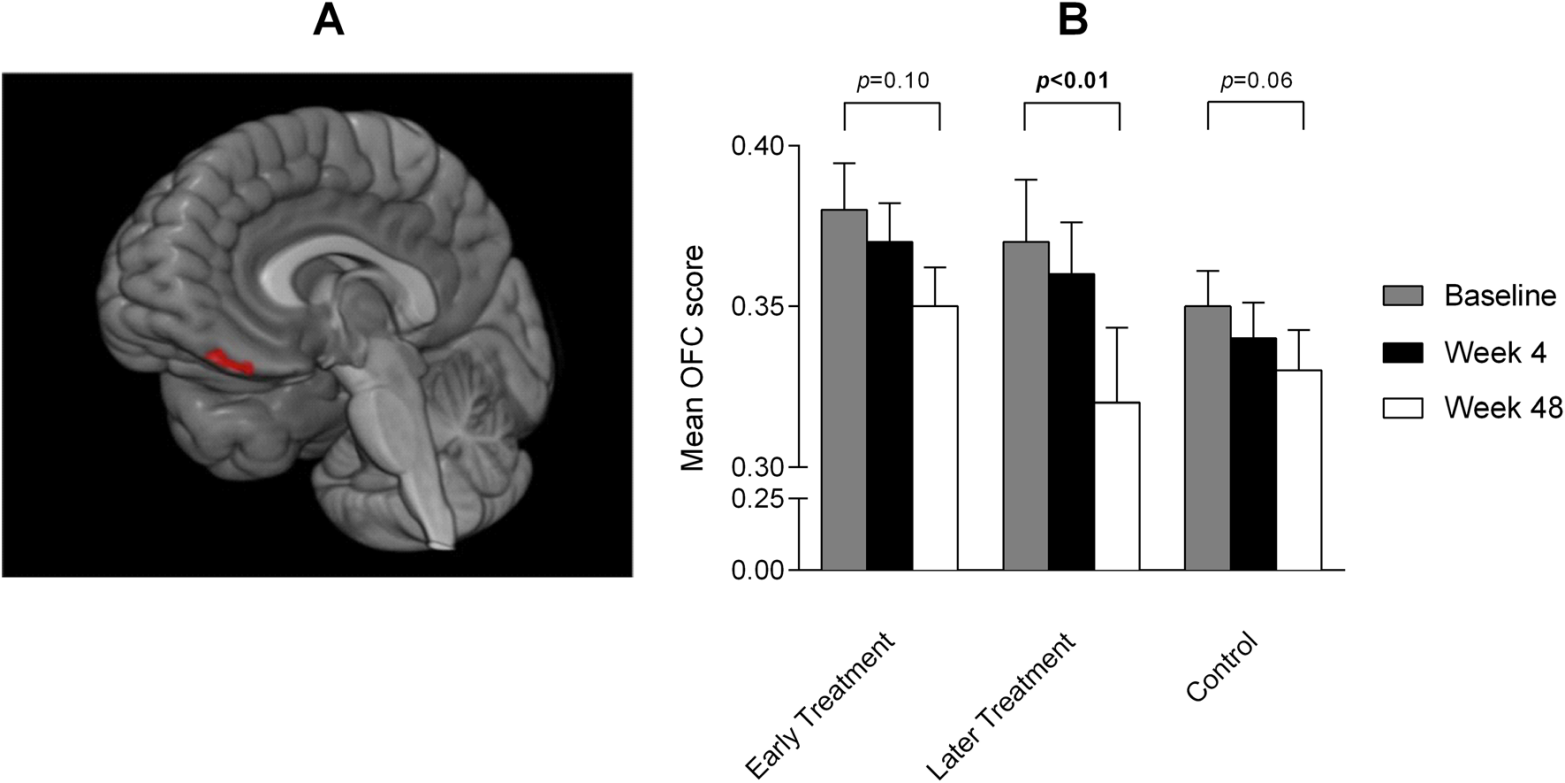
Medial orbitofrontal cortex and mean volume values. (**A**) Voxels showing a significantly larger decrease in gray matter volume at week 4 in relation to baseline in the later treatment arm than in the early treatment arm. These voxels were located at the medial orbitofrontal cortex, with the most significant finding (peak coordinate) at x = 6; y = 44; z = –32 (T = 4.46; *p*_(FWE)_ = 0.030). (**B**) Mean medial orbitofrontal cortex scores found prospectively. Upper bars represent the standard error of the mean. The Y axis is disproportionate to provide a more optimal presentation of outcomes. The statistical test applied was the longitudinal ANOVA for repeated measures. Bold *p* value denotes statistical significance. Abbreviations: OFC = orbitofrontal cortex.

Exploratory longitudinal voxel-wise analyses showed that gray matter had decreased significantly more in the later treatment arm than in the early treatment arm at week 4, specifically in the medial orbitofrontal cortex (*p*_[FWE]_ = 0.030) (Fig. 3A).

There was no significant difference between the 3 groups at week 48. Longitudinal intra-group progression of gray matter volume revealed a statistically significant decrease in the medial orbitofrontal cortex across the timepoints in the later treatment arm (*p* = 0.001), which was not observed in the early treatment arm (*p* = 0.101) or the control arm (*p* = 0.064) (Fig. 3B).

## Discussion

We did not observe significant differences in the cognitive status of men who had recently acquired HIV-1 infection (< 3 months), men whose estimated time since HIV-1 acquisition was longer than 6 months, or men without HIV infection in this small prospective observational trial. The presence of HAND is well established and accepted in PLWH^14^. However, the stage of infection when disorders first manifest is not well identified. Our data indicate that cognitive changes may be subtle during the initial stages of HIV-1 infection and that the neurobiological mechanisms underlying disruption of the CNS may not have developed sufficiently for cognitive disorders to manifest, as other investigators have suggested^25–27^. The correlation found between worse neuropsychological performance (NPZ-12 score) and longer time from diagnosis of HIV-1 infection would also support this hypothesis. In addition, the mean age of the study sample was not very high, and immunological parameters were mostly preserved. Also relevant is the fact that we did not detect significant cognitive improvement after 48 weeks of cART with INSTI, when both treatment groups were compared with untreated seronegative individuals. This effect could reasonably be anticipated due to the absence of baseline cognitive impairment in the HIV arms. In most studies that report cognitive improvement linked to initiation of cART, individuals had marked impairment before therapy was started^28^. Another explanation for not finding cognitive changes over time is the limited period covered in the follow-up study (12 months). It is also important to note that the most extensively used criteria to categorize cognitive impairment in HIV infection, the Frascati criteria, have been called into question owing to the potential for misclassification^29,30^. Cerebrospinal fluid analyses could have provided additional information about slight improvements in inflammatory or neurological biomarkers, thus complementing subtle cognitive changes not captured by neuropsychological assessments. However, unfortunately, analysis of cerebrospinal fluid was not included in the methodology of our study.

One of the strengths of this trial was that it assessed a variable range of functional measures. Such an approach is very unusual. In fact, in people with early infection we reveal significantly worse emotional status, which is not found in PLWH who initiate treatment later. We detected symptoms at 3 levels: depressive, anxiety, and perceived stress. Consistent with our data, other studies have reported high rates of depressive symptoms in people with recent/acute HIV-1 infection^31,32^. This finding could reasonably be explained as the initial psychological impact of a recent diagnosis of HIV infection. Alternatively, some authors have pointed out that these alterations could be related to the immune activation that manifests during acute/primary infection^32^. Importantly, we found that these symptoms resolved after 48 weeks on therapy. The improvement in emotional status could be associated with the process of emotionally adapting to the infection, or as a result of using cART. In any case, INSTI-containing therapy appeared not to have a negative impact on emotional status.

Another interesting aspect of our work was the inclusion of structural brain imaging in the study analyses. This methodology has previously been used in the field, with proven sensitivity for detection of subtle anatomical brain changes associated with HIV infection^33^. Brain imaging did not reveal differences in brain imaging outcomes between the groups at baseline. Again, this could be explained by the short duration of the infection in both HIV groups. Most of the studies revealing subcortical and cortical atrophy in PLWH covered longer times with HIV infection^34–37^. Of note, when we analyzed structural outcomes longitudinally, we found a greater decrease in the gray matter of the medial orbitofrontal area in the participants who started therapy later. Previous cross-sectional investigations report reductions in the gray matter of the orbitofrontal cortex in PLWH compared with non-PLWH individuals, thus supporting our finding^34,38^. The results we report were prospective and appear to capture the initial stage of onset of these structural changes. Indeed, the orbitofrontal area seems to be susceptible to damage caused by HIV. This area is known to play a role in adaptive and objective behavior and is associated with decision-making processes^39^. Longitudinal neuroimaging studies investigating healthy aging support that a certain loss of gray matter is normal over time^40^. Consistent with this observation, we found a trend towards reduced medial orbitofrontal cortex in all groups, although the differences were only statistically significant in individuals who began cART later. While INSTI have been reported to induce neurotoxicity through several molecular mechanisms in in vitro studies, such as altering lysosomal function leading to neuroinflammation^41^ or hyperexcitability of medial prefrontal cortex pyramidal neurons^42^, our data show that the longer-term effect of HIV-1 itself in the brain could be more harmful than potential INSTI-related neurotoxicity, at least to the extent that neuroimaging can reveal clinically significant findings.

Our study was subject to limitations. First, the sample size was small, and data from a reduced group of participants were not included in the prospective analyses because of the exclusion criteria. In addition, the sample only included MLWH, thus limiting the generalizability of the results to PLWH. In fact, different outcomes might be found in women living with HIV, in part due to anatomical differences in brain structure or other potential sex-related confounders. Second, we recorded a fairly high rate of illicit drug use, which could affect brain-related outcomes. Third, the results are mostly representative of the INSTI studied and may not therefore be applicable to other currently used agents in clinical practice (e.g., bictegravir or cabotegravir). Finally, cerebrospinal fluid was not evaluated. Data on this analytical parameter may have been able to explain outcomes connected with cognitive and brain imaging findings. The main reason for not including these evaluations was the intention not to enlarge the assessment burden for study participants, since evaluations were already very comprehensive. The fact that participants were assessed at 3 points also prevented this parameter from being used.

## Summary and conclusion

According to our findings, changes in CNS functioning in early stages of HIV infection appear to be subtle and do not seem to substantially affect cognition. However, functional status is already affected during the first 3 months after infection, and early initiation of cART with INSTI is beneficial for emotional status in particular. Regarding brain imaging status, we report that MLWH initiating cART later may experience a more marked decrease in medial orbitofrontal cortex volume over time, with likely negative repercussions for decision-making tasks.

## Supplementary Information

Supplementary Information.

## Data Availability

All study data are available from Fundació Lluita contra la SIDA (www.flsida.org) in line with local data protection laws. Anonymized data can be shared for academic and scientific purposes, always following required ethical procedures. Requests can be made by contacting the corresponding authors.
