# Supplementary material for: Effects of integrase inhibitor-based antiretroviral therapy on brain outcomes according to time since acquisition of HIV-1 infection
Source: Sci Rep. 2021 May 28;11:11289. doi: 10.1038/s41598-021-90678-6 (PMC8163778; doi:10.1038/s41598-021-90678-6)
Supplement: Supplementary file 1 — Supplementary Information. [file 41598_2021_90678_MOESM1_ESM.docx]

|  | **Early**  **Treatment**  **(n=12)** | **Later Treatment**  **(n=15)** | **Control**  **(n=15)** | ***p* Value *^a ; b^*** | ***d* Size *^a ; b^*** |
| --- | --- | --- | --- | --- | --- |
| **Attention/working memory** |  |  |  |  |  |
| Digit span forward (WAIS-IV)^1^ | 0.02 (1.12) | 0.00 (0.91) | -0.40 (0.76) | 0.253; 0.202 | 0.44; 0.46 |
| Digit span backward (WAIS-IV)^1^ | 0.28 (1.25) | 0.50 (1.04) | 0.30 (0.88) | 0.955; 0.589 | -0.02; 0.20 |
| **Information processing speed** |  |  |  |  |  |
| Total time (TMT-A)^2^ | 0.52 (0.76) | 0.78 (0.96) | 0.72 (0.68) | 0.474; 0.863 | -0.27; 0.07 |
| Written score (SDMT)^3^ | 0.21 (0.82) | 0.21 (1.04) | 0.44 (1.08) | 0.562; 0.565 | -0.23; -0.21 |
| **Motor function** |  |  |  |  |  |
| Dominant hand time (GPT)^4^ | 1.05 (0.61) | 0.68 (0.67) | 0.49 (0.79) | 0.056; 0.491 | 0.76; 0.25 |
| Non-dominant hand time (GPT)^4^ | 0.64 (0.85) | 0.40 (0.90) | 0.28 (0.83) | 0.278; 0.692 | 0.42; 0.13 |
| **Learning and verbal memory** |  |  |  |  |  |
| Total A list (CVLT-II)^5^ | 1.78 (0.84) | 1.01 (0.85) | 1.05 (0.99) | 0.062; 0.907 | 0.76; -0.04 |
| Long-term free recall (CVLT-II)^5^ | 1.04 (0.63) | 0.95 (0.69) | 0.94 (0.66) | 0.317; 0.777 | 0.15; 0.01 |
| **Verbal fluency** |  |  |  |  |  |
| Phonemic (Letter “p”)^6^ | -0.08 (0.69) | -0.17 (0.73) | -0.16 (0.75) | 0.789; 0.961 | 0.11; -0.01 |
| Semantic (Animals test)^7^ | -0.71 (0.92) | -0.26 (0.92) | -0.40 (0.66) | 0.330; 0.654 | -0.39; 0.17 |
| **Executive function** |  |  |  |  |  |
| Total time (TMT-B)^2^ | 0.36 (0.54) | 0.40 (0.51) | 0.42 (0.59) | 0.789; 0.896 | -0.10; -0.04 |
| Total moves (TOL)^8^ | 0.08 (1.00) | 0.02 (1.20) | 0.40 (1.19) | 0.460; 0.393 | -0.28; -0.31 |
| **Global** |  |  |  |  |  |
| NPZ-12 | 0.44 (0.45) | 0.37 (0.38) | 0.33 (0.37) | 0.488; 0.777 | 0.26; 0.10 |
| **Cognitive complaints, n (%)** | 2 (17) | 2 (13) | 2 (13) | 1; 1 | 0.112; 0 |
| **Cognitive impairment, n (%)** | 4 (33) | 4 (27) | 1 (7) | 0.139; 0.330 | 0.688; 0.557 |
| **HAND** | 4 (33) | 4 (27) | - | - | - |
| ANI | 4 (100) | 4 (100) | - | - | - |
| MND | 0 | 0 | - | - | - |
| HAD | 0 | 0 | - | - | - |

**Supplementary Table S1.** Specific cognitive scores at baseline.

Values are expressed as mean (standard deviation).

Continuous variables were compared using the independent *t* test. Categorical variables were compared using the Fisher exact test. Statistical significance was set at *p*<0.05.

*d* size was obtained using Cohen's *d*. Values are considered small when <0.20, medium when ≥0.50 and ≤0.80, and large when >0.80.

Abbreviations: ANI = asymptomatic neurocognitive impairment; CVLT-II = California Verbal Learning Test - Version II; GPT = Grooved Pegboard Test; HAD = HIV-associated dementia; HAND = HIV-associated neurocognitive disorder; MND = minor neurocognitive disorder; SDMT = Symbol Digit Modalities Test; TMT-A = Trail Making Test - Part A; TMT-B = Trail Making Test - Part B; WAIS-IV = Wechsler Adult Intelligence Scale - Version IV; TOL = Tower of London.

*^a^* Comparison between early treatment and control arms.

*^b^* Comparison between later treatment and control arms.

**References:**

1. Wechsler, D. *Wechsler Adult Intelligence Scale - Fourth Edition.* (Pearson, 2008).

2. Reitan, R. M. & Davison, L. A. *Clinical neuropsychology: current status and applications.* (John Wiley & Sons Inc, 1974).

3. Smith, A. *Symbol Digit Modalities Test*. (Western Psychological Services, 1973).

4. Reitan, RM and Wolfson, D. *The Halstead-Reitan Extended Neuropsychological Test Battery*. (Neuropsychology Press, 1985).

5. Delis, D., Kramer, J., Kaplan, E. & Ober, B. *California Verbal Learning Test - Second Edition*. (Pearson, 2000).

6. Artiola i Fortuny, L., Romo, D., Heaton, R. & Pardee, R. *Manual de normas y procedimientos para la batería neuropsicológica en español*. (M Press, 1999).

7. Kertesz, A. *Western Aphasia Battery.* (The Psychological Corp, 1982).

8. Culbertson, W. C. & Zillmer, E. A. *The Tower of London, Drexel University, research version: Examiner’s manual*. (Multi-Health Systems, 2001).

|  |  | **Early**  **Treatment**  **(n=12)** | | **Later**  **Treatment**  **(n=15)** | | **Control**  **(n=15)** | |  |  |
| --- | --- | --- | --- | --- | --- | --- | --- | --- | --- |
|  | Change from baseline | No. of subjects | Mean (SD) | No. of subjects | Mean (SD) | No. of subjects | Mean (SD) | *p* Value *^a ; b^* | *d* Size *^a ; b^* |
| **Attention/working memory** |  |  |  |  |  |  |  |  |  |
| Digit span forward (WAIS-IV) |  |  |  |  |  |  |  |  |  |
|  | Week 4 | 12 | 0.09 (0.84) | 15 | 0.45 (0.71) | 14 | 0.60 (1.15) | 0.218; 0.681 | -0.48; -0.15 |
|  | Week 48 | 11 | 0.15 (0.75) | 15 | 0.22 (0.86) | 10 | 0.59 (1.16) | 0.318; 0.371 | 0.44; -0.38 |
| Digit span backward (WAIS-IV) |  |  |  |  |  |  |  |  |  |
|  | Week 4 | 12 | -0.02 (1.25) | 15 | 0.12 (0.81) | 14 | -0.02 (0.90) | 0.993; 0.630 | 0.01; 0.18 |
|  | Week 48 | 11 | 0.10 (1.36) | 15 | 0.30 (0.88) | 10 | -0.32 (0.80) | 0.408; 0.085 | 0.36; 0.51 |
| **Information processing speed** |  |  |  |  |  |  |  |  |  |
| Total time (TMT-A) |  |  |  |  |  |  |  |  |  |
|  | Week 4 | 12 | 0.17 (0.63) | 15 | 0.19 (0.51) | 14 | -0.01 (0.76) | 0.342; 0.923 | 0.22; 0.32 |
|  | Week 48 | 11 | 0.06 (0.83) | 15 | 0.50 (0.49) | 10 | 0.34 (0.30) | 0.337; 0.350 | -0.42; 0.23 |
| Written score (SDMT) |  |  |  |  |  |  |  |  |  |
|  | Week 4 | 12 | 0.02 (0.94) | 15 | 0.35 (0.72) | 14 | 0.32 (0.64) | 0.502; 0.395 | -0.38; 0.03 |
|  | Week 48 | 11 | 0.38 (0.58) | 15 | 0.11 (0.60) | 10 | 0.12 (0.84) | 0.433; 0.985 | 0.35; -0.01 |
| **Motor function** |  |  |  |  |  |  |  |  |  |
| Dominant hand time (GPT) |  |  |  |  |  |  |  |  |  |
|  | Week 4 | 12 | 0.08 (0.51) | 15 | 0.29 (0.52) | 14 | 0.05 (0.61) | 0.883; 0.259 | 0.05; 0.41 |
|  | Week 48 | 11 | 0.03 (0,43) | 15 | 0.26 (0.61) | 10 | 0.22 (0.26) | 0.265; 0.823 | -0.51; 0.10 |
| Non-dominant hand time (GPT) |  |  |  |  |  |  |  |  |  |
|  | Week 4 | 12 | 0.15 (0.58) | 15 | 0.02 (0.74) | 14 | 0.34 (0.64) | 0.438; 0.233 | -0.30; -0.43 |
|  | Week 48 | 11 | 0.23 (0.76) | 15 | 0.30 (0.68) | 10 | 0.50 (0.57) | 0.385; 0.453 | -0.49; -0.37 |
| **Learning and verbal memory** |  |  |  |  |  |  |  |  |  |
| Total A list (CVLT-II) |  |  |  |  |  |  |  |  |  |
|  | Week 4 | 12 | -0.06 (0.65) | 15 | 0.15 (0.70) | 14 | 0.13 (0.80) | 0.511; 0.950 | -0.26; 0.01 |
|  | Week 48 | 11 | -0.29 (0.80) | 15 | 0.43 (0.77) | 10 | 0.41 (0.52) | **0.031**; 0.934 | -0.99; 0.03 |
| Long-term free recall (CVLT-II) |  |  |  |  |  |  |  |  |  |
|  | Week 4 | 12 | -0.04 (0.56) | 15 | -0.14 (0.92) | 14 | -0.35 (0.79) | 0.284; 0.518 | 0.44; 0.25 |
|  | Week 48 | 11 | -0.09 (0.53) | 15 | 0.16 (0.72) | 10 | 0.10 (0.39) | 0.370; 0.793 | -0.39; 0.12 |
| **Verbal fluency** |  |  |  |  |  |  |  |  |  |
| Phonemic (Letter “p”) |  |  |  |  |  |  |  |  |  |
|  | Week 4 | 12 | 0.05 (0.47) | 15 | 0.34 (0.62) | 14 | 0.05 (0.73) | 0.992; 0.270 | -0.02; 0.40 |
|  | Week 48 | 11 | 0.01 (0.73) | 15 | 0.30 (0.85) | 10 | 0.06 (0.92) | 0.889; 0.500 | -0.06; 0.29 |
| Semantic (Animals test) |  |  |  |  |  |  |  |  |  |
|  | Week 4 | 12 | 0.21 (0.48) | 15 | -0.26 (0.73) | 14 | -0.06 (0.71) | 0.274; 0.457 | 0.42; -0.28 |
|  | Week 48 | 11 | 0.51 (0.61) | 15 | 0.06 (1.05) | 10 | -0.11 (0.58) | **0.029**; 0.635 | 1; 0.27 |
| **Executive function** |  |  |  |  |  |  |  |  |  |
| Total time (TMT-B) |  |  |  |  |  |  |  |  |  |
|  | Week 4 | 12 | 0.00 (0.37) | 15 | -0.04 (0.68) | 14 | -0.15 (0.97) | 0.584; 0.724 | 0.20; 0.13 |
|  | Week 48 | 11 | 0.07 (0.45) | 15 | 0.30 (0.57) | 10 | 0.11 (0.62) | 0.877; 0.442 | -0.07; 0.35 |
| Total moves (TOL) |  |  |  |  |  |  |  |  |  |
|  | Week 4 | 12 | 0.18 (0.69) | 15 | 0.58 (0.92) | 14 | 0.52 (1.18) | 0.383; 0.897 | -0.34; 0.05 |
|  | Week 48 | 11 | -0.05 (0.77) | 15 | 0.70 (0.97) | 10 | 0.97 (0.88) | **0.011**; 0.498 | -1.19; -0.28 |
| **Global** |  |  |  |  |  |  |  |  |  |
| NPZ-12 |  |  |  |  |  |  |  |  |  |
|  | Week 4 | 12 | 0.06 (0.20) | 15 | 0.14 (0.25) | 14 | 0.11 (0.28) | 0.602; 0.789 | -0.24; 0.07 |
|  | Week 48 | 11 | 0.08 (0.26) | 15 | 0.31 (0.28) | 10 | 0.25 (0.19) | 0.117; 0.538 | -0.17; 0.25 |

**Supplementary Table S2.** Change in specific cognitive scores.

Values are expressed as mean (standard deviation). The treatment and control arms were compared using the independent *t* test. Statistical significance was set at *p*<0.05. Bold values denote statistical significance.

*d* size was obtained using Cohen's *d*. Values are considered small when <0.20, medium when ≥0.50 and ≤0.80, and large when >0.80.

Abbreviations: CVLT-II = California Verbal Learning Test - Version II; GPT = Grooved Pegboard Test; SDMT = Symbol Digit Modalities Test; TMT-A = Trail Making Test - Part A; TMT-B = Trail Making Test - Part B; WAIS-IV = Wechsler Adult Intelligence Scale - Version IV; TOL = Tower of London.

*^a^* Comparison between the early treatment and control arms.

*^b^* Comparison between the later treatment and control arms.

|  | **Early Treatment**  **(n=12)** | **Later Treatment**  **(n=15)** | **Control**  **(n=15)** | ***p* Value *^a ; b^*** | ***d* Size *^a ; b^*** |
| --- | --- | --- | --- | --- | --- |
| **CNS adverse events** |  |  |  |  |  |
| Total | 13 (11.56) | 11.71 (7.90) | 9.20 (7.43) | 0.311; 0.385 | 0.39; 0.32 |
| **Daily functioning** |  |  |  |  |  |
| Total impaired areas | 0.25 (0.62) | 0.80 (1.56) | 0.60 (1.05) | 0.320; 0.685 | -0.38; 0.15 |
| **Emotional status** |  |  |  |  |  |
| Depressive symptoms | 5.08 (2.77) | 4.13 (2.56) | 2.53 (2.03) | **0.011**; 0.068 | 1.04; 0.67 |
| Anxiety symptoms | 9.08 (4.38) | 7.20 (3.01) | 5.47 (3.42) | **0.024**; 0.151 | 0.90; 0.52 |
| Perceived stress | 17.33 (7.25) | 14.93 (7.93) | 10.40 (5.75) | **0.010**; 0.084 | 1.04; 0.64 |
| **Quality of life** |  |  |  |  |  |
| Global dimension | 3.16 (0.39) | 3.13 (0.64) | 3.33 (0.48) | 0.345; 0.344 | -0.37; -0.34 |

**Supplementary Table S3.** Functional scores at baseline examination.

Values are expressed as mean (standard deviation).

The treatment and control arms were compared using the independent *t* test. Statistical significance was set at *p*<0.05. Bold values denote statistical significance.

*d* size was obtained using Cohen's *d*. Values are considered small when <0.20, medium when ≥0.50 and ≤0.80, and large when >0.80.

Abbreviations: CNS = central nervous system.

*^a^* Comparison between the early treatment and control arms.

*^b^* Comparison between the later treatment and control arms.
